# The myristoylated amino-terminus of an Arabidopsis calcium-dependent protein kinase mediates plasma membrane localization

**DOI:** 10.1007/s11103-013-0061-0

**Published:** 2013-04-23

**Authors:** Sheen X. Lu, Estelle M. Hrabak

**Affiliations:** 1 Department of Molecular, Cellular & Biomedical Sciences, University of New Hampshire, Durham, NH 03824 USA; 2 Present Address: Department of Molecular, Cellular and Developmental Biology, University of California, Los Angeles, CA 09905 USA

**Keywords:** Myristoylation, Acylation, Calcium, Kinase, Membrane targeting, Subcellular localization

## Abstract

Calcium-dependent protein kinases (CDPK) are a major group of calcium-stimulated kinases found in plants and some protists. Many CDPKs are membrane-associated, presumably because of lipid modifications at their amino termini. We investigated the subcellular location and myristoylation of AtCPK5, a member of the Arabidopsis CDPK family. Most AtCPK5 was associated with the plasma membrane as demonstrated by two-phase fractionation of plant microsomes and by in vivo detection of AtCPK5-GFP fusion proteins. AtCPK5 was a substrate for plant *N*-myristoyltransferase and myristoylation was prevented by converting the glycine at the proposed site of myristate attachment to alanine (G2A). In transgenic plants, a G2A mutation completely abolished AtCPK5 membrane association, indicating that myristoylation was essential for membrane binding. The first sixteen amino acids of AtCPK5 were sufficient to direct plasma membrane localization. In addition, differentially phosphorylated forms of AtCPK5 were detected both *in planta* and after expression of AtCPK5 in a cell-free plant extract. Our results demonstrate that AtCPK5 is myristoylated at its amino terminus and that myristoylation is required for membrane binding.

## Introduction

Calcium is a ubiquitous signaling molecule in plants (Hashimoto and Kudla 2011). Transient cytosolic calcium signals are generated in response to environmental signals or stresses such as light, cold, pathogens, or drought, as well as in response to internal stimuli, and these signals are decoded by various calcium-binding proteins (DeFalco et al. 2010). Since calcium has limited diffusion potential in the cytosol (Allbritton et al. 1992), rapid and efficient calcium detection occurs when calcium sensor proteins are located at cell membranes in close proximity to sites of calcium influx.

Proteins in the calcium-dependent protein kinase (CDPK) family have characteristics that enable them to be effective transducers of cytosolic calcium fluxes. All CDPKs have a typical serine/threonine kinase catalytic domain fused via a regulatory autoinhibitory domain to a calmodulin-like domain that directly binds calcium (Cheng et al. 2002; Hrabak et al. 2003; Mehlmer et al. 2010). Calcium binding to the calmodulin-like domain induces intramolecular conformational changes that lead to rapid and calcium-specific activation of the catalytic domain (Huang et al. 1996). CDPKs also contain a highly divergent amino-terminal variable domain that is critical for correct subcellular localization of the enzyme (Martin and Busconi 2000; Lu and Hrabak 2002; Dammann et al. 2003; Raices et al. 2003; Chehab et al. 2004; Gargantini et al. 2006) and may also play a role in substrate recognition (Ito et al. 2010).

Although not found in metazoans, CDPKs have been identified in all plants examined to date, including mosses, liverworts, green algae, gymnosperms and angiosperms, and are also found in the apicomplexan protists (Zhang and Choi 2001). Most plants encode multiple CDPK isoforms. For example, 34 genes encoding CDPKs have been identified in the *Arabidopsis thaliana* genome (Hrabak et al. 2003), while there are 29 in rice (Asano et al. 2005) and at least 21 in *Medicago truncatula* (Gargantini et al. 2006). CDPK isoforms may vary in substrate specificity, enzyme kinetics, expression pattern, or subcellular location (Hrabak et al. 2003; Harper et al. 2004) and are involved in processes such as carbon and nitrogen metabolism (Douglas et al. 1998; Asano et al. 2002), plant growth and development (Ivashuta et al. 2005; Gargantini et al. 2006; Yoon et al. 2006), defense against pathogens (Romeis et al. 2001; Freymark et al. 2007; Kobayashi et al. 2007), and responses to hormones and abiotic stresses (Abbasi et al. 2004; Ludwig et al. 2005; Szczegielniak et al. 2005; Ma and Wu 2007; Zhu et al. 2007; Franz et al. 2011).

Many CDPKs are membrane associated although they do not contain recognizable transmembrane domains. In Arabidopsis, 10 of the 34 CDPKs have been localized to the plasma membrane, peroxisome, or endoplasmic reticulum, while two are predominantly cytosolic (Lu and Hrabak 2002; Dammann et al. 2003; Rodriguez Milla et al. 2006; Zhu et al. 2007; Coca and San Segundo 2010; Mehlmer et al. 2010). Membrane binding of CDPKs is likely mediated by acylation of the amino-terminal variable domain. Myristoylation was first demonstrated for a zucchini CDPK (Ellard-Ivey et al. 1999) and has subsequently been reported for CDPKs from other species. In Arabidopsis, the variable domain of AtCPK2 is myristoylated and this modification is required for membrane association (Lu and Hrabak 2002). Similar results have been reported for CDPKs from rice (Martin and Busconi 2000), ice plant (Chehab et al. 2004), potato (Raices et al. 2001; Raices et al. 2003), and tomato (Rutschmann et al. 2002).

Many myristoylated proteins are known or are predicted to be involved in cellular signaling pathways (Boisson et al. 2003; Maurer-Stroh et al. 2004; Resh 2004), and myristoylation is often required for correct protein function. For example, in Arabidopsis, myristoylation of the SOS3 calcium-binding protein is required for salt tolerance (Ishitani et al. 2000), BON1/CPN1 myristoylation is required for normal plant growth (Li et al. 2010), and SnRK1 myristoylation affects the catalytic activity of this kinase and its role in shoot meristem development (Pierre et al. 2007).

Protein myristoylation is catalyzed by myristoyl-CoA:protein *N*-myristoyltransferase (NMT; Rajala et al. 2000; Farazi et al. 2001). Following removal of the initiator methionine, NMT catalyzes the formation of a stable amide bond between myristate (C14:0) and the exposed amino-terminal glycine (Gly-2) residue of a substrate protein. In addition to an absolute requirement for Gly-2, the amino acids immediately following Gly-2 play critical roles in determining whether a protein can be a substrate for NMT (Towler et al. 1988; Rocque et al. 1993; Utsumi et al. 2004). Computer algorithms developed to predict protein myristoylation (Maurer-Stroh et al. 2002b; Bologna et al. 2004; Podell and Gribskov 2004) indicate that many CDPKs have putative *N*-myristoylation sites at their amino termini.

In this study, we investigate protein modifications and subcellular location of a CDPK from *Arabidopsis thaliana*, AtCPK5. We demonstrate that AtCPK5 is myristoylated in vitro, although this was not predicted by all of the available myristoylation programs, and that the majority of the AtCPK5 protein in plant cells is associated with the plasma membrane. We also present evidence that phosphorylation of AtCPK5 occurs in plant extracts. Mutation of Gly-2 prevents myristoylation and abolishes membrane association of AtCPK5 in plants. Finally, we demonstrate that the first 16 amino acids of AtCPK5 are sufficient to direct plasma membrane targeting.

## Experimental procedures

### Plasmid constructs

The pCPK5-16aa-GUS construct contained a 1,417 bp genomic DNA fragment followed in-frame by the β-glucuronidase (GUS) coding sequence from the *E. coli uidA* gene and the *nos* terminator. The 1,417 bp Arabidopsis genomic DNA fragment (Arabidopsis gene At4g35310) consisted of 50 nucleotides of coding sequence preceded by the 449 bp *CPK5* untranslated leader (containing a 224 bp intron) and 918 bp of non-transcribed sequence, presumed to contain the promoter region. The GUS coding sequence and the *nos* terminator were from pBI101 (Clontech, Mountain View, CA, USA). For plant transformation, this entire region was cloned into pBIN19 (Bevan 1984) to create pCPK5-16aa-GUS. The first 16 amino acids of AtCPK5 are MGNSCRGSFKDKLDEG. Mutagenesis of the glycine codon (GGC) at position 2 to alanine (GCC) was performed with the QuikChange Site-Directed Mutagenesis kit (Stratagene, La Jolla, CA, USA) according to the manufacturer’s instructions to create pCPK5-G2A-GUS. The presence of the G2A mutation was confirmed by DNA sequencing. For constructs pCPK5-16aa-GFP and pCPK5-G2A-GFP, the GUS coding sequence in pCPK5-16aa-GUS and pCPK5-G2A-GUS was replaced with the coding sequence for soluble-modified red-shifted green fluorescent protein (smRS-GFP, Davis and Vierstra 1998).

### Plant transformation and growth conditions

*Arabidopsis thaliana* (ecotype Columbia) plants were transformed by the floral dip method (Clough and Bent 1998) and transgenics were selected on solidified Murashige and Skoog basal medium with Gamborg’s B-5 vitamins (Sigma, St. Louis, MO, USA) and 0.1 % (w/v) sucrose, pH 5.7, containing 50 mg/L kanamycin. Kanamycin-resistant plants were confirmed to contain the transgene using a rapid PCR method (Klimyuk et al. 1993).

### Membrane isolation and aqueous two-phase partitioning

Seeds from transgenic plants were surface-sterilized and grown in liquid Murashige and Skoog basal medium with Gamborg’s B-5 vitamins and 1 % (w/v) sucrose, pH 5.7, at 21 °C with an 18 h photoperiod. Aeration was maintained on a rotary shaker at 120 rpm. Microsomal membranes were prepared using a modification of a previously described procedure (Schaller and DeWitt 1995). All homogenization and fractionation steps were conducted on ice or in a cold room with prechilled buffers and equipment. All buffers contained protease inhibitor cocktail (Roche, Indianapolis, IN, USA). Two-week-old plants were ground in homogenization buffer (50 mM Tris–HCl, pH 8.2, 20 % [v/v] glycerol, 2 mM EDTA; 1 ml/g fresh weight) with a mortar and pestle. The homogenates were cleared by filtration through Miracloth (Calbiochem, La Jolla, CA, USA) followed by centrifugation at 5,000×*g* for 5 min. Microsomal membranes were isolated by centrifugation of the supernatant for 30 min at 125,000×*g*. Membrane pellets were resuspended in SPK buffer (0.33 M sucrose, 5 mM KPO_4_, and 3 mM KCl, pH 7.8) using a ground glass homogenizer. Aqueous two-phase partitioning to enrich for plasma membrane vesicles was conducted essentially as described (Larsson et al. 1987). Resuspended microsomes were added to a 6.3 % (w/w) DextranT500/PEG3350 phase mixture prepared in SPK buffer and the phases were separated at 1,000×*g* for 5–10 min. Both upper and lower phases were repartitioned twice with fresh lower or upper phase, respectively. The final upper and lower phases were diluted separately in 10 mM Tris–HCl (pH 7.0) containing 1 mM EDTA and 1 mM EGTA. Phase-partitioned membranes were collected by centrifugation at 125,000×*g* for 30 min and resuspended in equal volumes of SPK buffer.

### AtCPK5 antibodies

AtCPK5 rabbit polyclonal antiserum was raised against a fusion protein containing the first 50 amino acids of AtCPK5 fused to the carboxyl terminus of glutathione S-transferase. The construct was made in pGEX-KT (Hakes and Dixon 1992). Recombinant protein expressed in *E. coli* was purified on a glutathione-agarose matrix (Amersham Bioscience, Piscataway, NJ, USA) and injected into female New Zealand White rabbits. Crude serum was precipitated with 50 % ammonium sulfate and redissolved in phosphate-buffered saline (PBS) [137 mM NaCl, 4.3 mM Na_2_HPO_4_.7H_2_O, 1.5 mM KH_2_PO_4_ 2.7 mM KCl]. An affinity matrix for purifying AtCPK5 antibodies was prepared by cloning the *AtCPK5* cDNA into pRSET-A (Invitrogen, Carlsbad, CA, USA), purifying the expressed proteins on His-Select nickel affinity gel (Sigma), and crosslinking the purified AtCPK5-6His to Affi-Gel 15 (Bio-Rad Laboratories, Hercules, CA, USA). After binding of the partially purified antibody to this matrix, AtCPK5-specific antibodies were eluted with 0.1 M glycine (pH 2.7) and immediately neutralized with 0.1 volume of 1 M Tris–HCl (pH 8.0), followed by dialysis against PBS. Antibody was used at a 1000-fold dilution.

### Immunoblot analysis

For SDS-PAGE, samples were mixed with 3× Blue Loading Buffer (New England Biolabs, Beverly, MA, USA) and incubated at 37 °C for 30 min. After electrophoresis at 4 °C, proteins were electroblotted to PVDF membrane (Millipore, Bedford, MA, USA) and immunodetection was performed as described previously (Lu and Hrabak 2002). Antibodies for detection of specific membranes were: anti-H^+^-ATPase (DeWitt and Sussman 1995) for plasma membrane (1:10,000), anti-BiP (Hofte et al. 1992) for endoplasmic reticulum (1:1,000), and anti-β–ATPase D (Luethy et al. 1993) for mitochondrial membranes (1:100). Membranes were stripped between detections following the manufacturer’s instructions. Thylakoid membranes were identified by spectrophotometric detection of chlorophyll a and b (Lichtenthaler 1987).

### In vitro myristoylation assay

*AtCPK5* cDNA was inserted into pBluescript II KS (Stratagene) and expressed under control of the vector’s T7 promoter. The transcript produced from this clone contains 38 bp of sequence from the vector and 18 bp of the *AtCPK5* leader preceding the AtCPK5 translation initiation codon. There is no alternate start codon in the upstream sequence. Myristoylation assays were performed with the TnT Coupled Transcription/Translation Wheat Germ Extract System (Promega Corp., Madison, WI, USA) according to the manufacturer’s instructions. cDNAs encoding either wildtype or mutant AtCPK5 proteins were in vitro transcribed and translated in the presence of either 10 μCi of L-[^35^S]-methionine (1,000 Ci/mmol; Amersham) or 50 μCi of [9,10-^3^H]-myristic acid (54 Ci/mmol; Amersham Bioscience). Before use, the myristic acid was dried under nitrogen and resuspended by vortexing in DEPC-treated water at a concentration of 10 μCi/μl. Control reactions contained no plasmid. After 90 min incubation at 30 °C, reaction products were separated by SDS-PAGE and analyzed by fluorography with Entensify Universal Autoradiography Enhancer (PerkinElmer, Shelton, CT, USA).

### Dephosphorylation assay

Products from in vitro myristoylation assays (10 μl) or the upper phase from two phase partitioning (10 μl) were incubated with 5 units of calf intestinal alkaline phosphatase (Roche) for 15 min at 30 °C. Control reactions contained phosphatase inhibitor cocktail II (Sigma). The reactions were subsequently boiled in the presence of 3× Blue Loading Buffer, separated by SDS-PAGE, and AtCPK5 was visualized by immunoblotting.

### β-Glucuronidase assays

β-Glucuronidase (GUS) activity was determined with a fluorometric assay (Gallagher 1992). For CPK5-16aa-GUS plants, total membrane-bound GUS activity (measured in nmol min^−1^ mg^−1^) ranged from 1.1 × 10^4^ to 1.8 × 10^4^ (*n* = 7), while GUS activity from CPK5-G2A-GUS plants ranged from 1.0 × 10^3^ to 2.4 × 10^3^ (*n* = 6).

### Transient expression of AtCPK5-GFP fusion proteins

p35SBSYFP (Katiyar-Agarwal et al. 2006), pCPK5-16aa-GFP, or pCPK5-G2A-GFP were purified using a QIAprep Spin Miniprep Kit (Qiagen Inc., Valencia, CA, USA). Rosette leaves (0.8–1.2 cm in length) were collected from 3- to 4-week-old Arabidopsis plants and bombarded with 0.8 μg plasmid DNA coated on 480 ug of gold particles (1 μm; Sigma) using a PDS-1000/He delivery system (Bio-Rad). The distance between the stop screen and the leaves was ~9 cm and a helium pressure of 650 psi was employed. Bombarded leaves were incubated in water at room temperature for 4–5 h. Images were collected using a SP2 confocal microscope (Leica Microsystems, Inc., Bannockburn, IL, USA), analyzed using MetaMorph 4.5 software (Universal Imaging Corp., Downingtown, PA, USA), and processed with Photoshop (Adobe Systems Inc., San Jose, CA, USA).

## Results

### AtCPK5 is associated with the plasma membrane

**Fig. 1:**
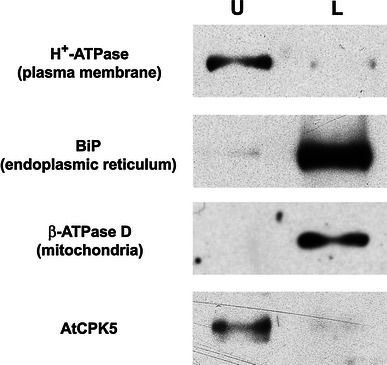
AtCPK5 is localized to the plasma membrane. Membranes from wildtype Arabidopsis plants were fractionated by aqueous two-phase partitioning. Equal proportions of the upper and lower phases were separated by SDS-PAGE, transferred to PVDF membranes, and analyzed sequentially using antibodies specific for AtCPK5 and for membrane marker proteins. *U* upper phase (enriched for plasma membrane); *L* lower phase (enriched for intracellular membranes)

Several Arabidopsis CDPKs are known to be membrane bound (Lu and Hrabak 2002; Dammann et al. 2003; Choi et al. 2005; Coca and San Segundo 2010; Mehlmer et al. 2010) although AtCPK5 has not been studied previously. To determine whether AtCPK5 was associated with the plasma membrane or with intracellular membranes, aqueous two-phase partitioning was used to fractionate microsomes from wildtype plants. This technique enriches plasma membrane vesicles in the upper phase while other intracellular membranes partition to the lower phase (Larsson et al. 1987). After phase separation, both phases were analyzed by immunoblotting to detect AtCPK5 protein and protein markers for specific membranes (Fig. 1). AtCPK5 was highly enriched in the upper phase, similar to the H^+^-ATPase plasma membrane marker. As expected, intracellular membrane marker proteins were enriched in the lower phase. These results indicate that AtCPK5 is associated with the plasma membrane but does not exclude the possibility that some AtCPK5 protein may be located in the cytosol.

### AtCPK5 is myristoylated in vitro

**Fig. 2:**
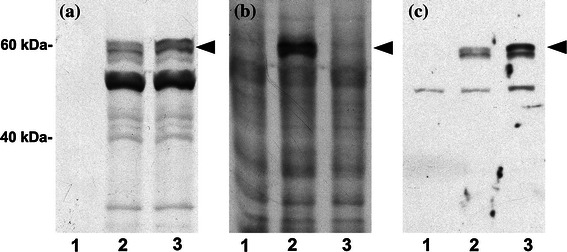
AtCPK5 is myristoylated in vitro and a G2A mutation prevents myristoylation. In a wheat germ coupled transcription and translation system, AtCPK5 protein (*lane 2*) was expressed in the presence of either **a** [^35^S]methionine; **b** [^3^H]myristic acid; or **c** no radiolabel, followed by SDS-PAGE and fluorography (**a** and **b**) or immunoblotting (**c**). Parallel experiments were performed on AtCPK5 protein containing a glycine-2 to alanine mutation (*lane 3*). Negative control reactions (*lane 1*) contained no plasmid template for gene expression. *Arrows* indicate AtCPK5 protein

To investigate whether the amino terminus of AtCPK5 contains a myristoylation consensus sequence, two myristoylation prediction programs were queried: Myristoylator (http://us.expasy.org/tools/myristoylator/myristoylator-ref.html; Bologna et al. 2004) and NMT Predictor (http://mendel.imp.ac.at/myriatate/SUPLpredictor.htm; Maurer-Stroh et al. 2002a). Myristoylator predicted myristoylation of AtCPK5 while NMT Predictor did not. To determine whether AtCPK5 could be myristoylated in vitro, we used a cell-free transcription/translation system from wheat germ that is known to contain NMT activity (Ellard-Ivey et al. 1999; Lu and Hrabak 2002). Both wildtype AtCPK5 protein and its glycine-2 to alanine (G2A) mutant were tested. Reactions included either [^35^S]-methionine for detection of total protein synthesis or [^3^H]-myristic acid for detection of myristoylated proteins. For both DNA templates, the major product of the [^35^S]-methionine-labeled reaction was a protein doublet near the predicted mass of 62.1 kDa (Fig. 2a). [^3^H]-myristate was incorporated into a 62 kDa product when the wildtype AtCPK5 protein was expressed but not when template DNA containing the G2A mutation was used (Fig. 2b). The identity of these radiolabeled proteins as AtCPK5 was confirmed by immunoblot analysis with AtCPK5 antibody, which recognizes the unique amino terminus of AtCPK5 (Fig. 2c). The 50 kDa protein recognized by the AtCPK5 antibodies represents non-specific binding to a wheat germ protein since it was present in a control reaction that contained no DNA template (Fig. 2c, lane 1). The smaller, non-myristoylated proteins routinely observed in [^35^S]methionine-labeled reactions may be alternative translation products from the AtCPK5 constructs or AtCPK5 degradation products (Fig. 2a). These results indicate that the AtCPK5 protein can be myristoylated in vitro and that Gly-2 is essential for myristoylation.

### AtCPK5 is phosphorylated in plants and in wheat germ extract

**Fig. 3:**
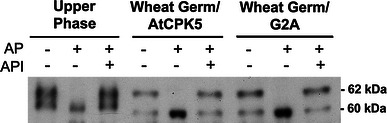
AtCPK5 is phosphorylated when expressed in planta or in vitro. The upper phase from two-phase partitioning of Arabidopsis membranes (*left*), cell-free wheat germ extract following in vitro expression of AtCPK5 (*center*), or cell-free wheat germ extract following expression of the AtCPK5-G2A mutant protein (*right*) were incubated with calf intestinal alkaline phosphatase (AP) and alkaline phosphatase inhibitor (API). The reaction products were separated by SDS-PAGE and AtCPK5 was detected by immunoblotting with AtCPK5 antibody

We noticed that AtCPK5 was often detected as a doublet of approximately 60 and 62 kDa whether the protein source was Arabidopsis plant extracts (Fig. 1) or wheat germ expression extracts (Fig. 2). This doublet was observed for both wildtype AtCPK5 and the G2A mutant protein, indicating that the second band is not due to the effects of myristoylation. Phosphorylation can retard the migration of proteins, so we examined whether the 62 kDa species represented a phosphorylated form of AtCPK5. Three different protein samples were tested: (1) the upper phase after aqueous two-phase partitioning of wildtype plant extracts, (2) wheat germ transcription/translation reactions expressing wildtype AtCPK5, or (3) wheat germ transcription/translation reactions expressing the G2A-mutated AtCPK5. Protein samples were treated with alkaline phosphatase in the presence or absence of alkaline phosphatase inhibitor. Alkaline phosphatase treatment resulted in the disappearance of the 62 kDa species (Fig. 3) and an increase in abundance of the 60 kDa species. In the presence of both alkaline phosphatase and alkaline phosphatase inhibitor, both the 60 and 62 kDa species were detected. Slight variations in migration are likely due to differences in buffer composition of the samples. These results indicate that phosphorylated AtCPK5 is present in plant extracts, that phosphorylation also occurs during in vitro synthesis of AtCPK5 in a cell-free extract, and that myristoylation and membrane association are not required for phosphorylation of AtCPK5.

### The first 16 amino acids of AtCPK5 are sufficient for plasma membrane localization

Correct subcellular targeting of acylated proteins from metazoans and yeast often requires only a short region at the amino-terminus of the protein (Di Paolo et al. 1997; Gillen et al. 1998; Alsheimer et al. 2000). Previously, we demonstrated that the amino terminus of a different Arabidopsis CDPK, AtCPK2, was sufficient for its correct targeting to the endoplasmic reticulum (Lu and Hrabak 2002). To determine whether the amino terminal region of AtCPK5 was sufficient for its membrane targeting, transgenic plants were produced that expressed the first 16 amino acids of AtCPK5 fused to the β-glucuronidase reporter protein (AtCPK5-16aa-GUS) under control of the native *AtCPK5* promoter. For comparison, plants were transformed with the T-DNA from pBI121 in which the GUS protein is expressed from the 35S promoter. To determine the relative distribution of GUS protein between cytosol and membranes, plant extracts were centrifuged to separate microsomes from soluble proteins as described previously and GUS activity in these two fractions was measured with a sensitive fluorometric assay. As expected, in control plants transformed with T-DNA from pBI121, the majority of the GUS protein was soluble and only a small amount (2–3 %) was membrane associated, probably due to non-specific membrane binding or trapping of the abundant GUS protein in membrane vesicles during preparation of extracts. In AtCPK5-16aa-GUS plant extracts, the majority (78 ± 3 %) of the GUS enzyme activity was in the microsomal fraction, indicating that most of the AtCPK5-GUS fusion protein was membrane associated and that the first 16 amino acids of AtCPK5 are sufficient for membrane targeting.

**Fig. 4:**
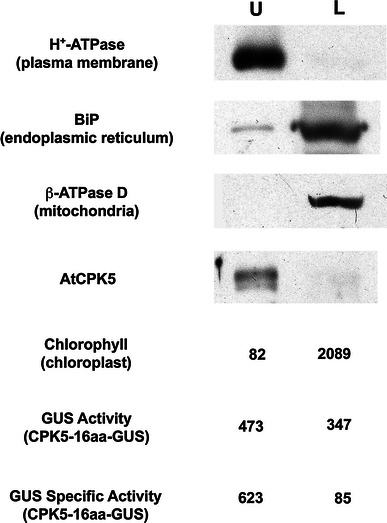
The first sixteen amino acids of AtCPK5 are sufficient for plasma membrane localization. Microsomal membranes from CPK5-16aa-GUS transgenic plants (expressing GUS protein preceded by the first 16 amino acids of AtCPK5) were analyzed by aqueous two-phase partitioning. Equal proportions of the upper and lower phases were separated by SDS-PAGE and assayed by immunoblotting with specific antibodies. Chlorophyll absorbance (ng/ml) was measured spectrophotometrically. The GUS enzyme was assayed fluorometrically and is expressed as activity (μmol/min/ml) and as specific activity (μmol/min/mg protein). *U* upper phase, *L* lower phase

To determine whether the CPK5-16aa-GUS fusion protein was targeted to the plasma membrane similar to wildtype AtCPK5 (Fig. 1), the microsomal fraction was used for aqueous two-phase partitioning. Both native AtCPK5 protein and the plasma membrane marker were enriched in the upper phase, while the markers for organellar membranes were concentrated in the lower phase (Fig. 4), confirming that the cellular membranes partitioned correctly. The location of the hybrid AtCPK5-16aa-GUS fusion protein was determined with a fluorometric assay for GUS activity. The plasma membrane-enriched upper phase contained more GUS activity per milliliter than the lower phase (Fig. 4). When expressed as specific activity to correct for the six-fold greater protein content in the lower phase, the difference between the amount of AtCPK5-16aa-GUS fusion protein in the two phases is apparent. These results indicate that the first 16 amino acids of AtCPK5 are sufficient to correctly target the soluble GUS protein to the plasma membrane.

### The G2A mutation abolishes AtCPK5 membrane association in plants

To address the importance of AtCPK5 myristoylation in membrane binding in plants, we examined the effect of mutation of the myristoylation site on membrane association. Site-directed mutagenesis was used to convert the second codon of pCPK5-16aa-GUS from glycine to alanine (G2A) to create pCPK5-G2A-GUS. Extracts from transgenic plants expressing the CPK5-G2A-GUS fusion protein were separated into soluble and microsomal fractions by ultracentrifugation. GUS enzyme activity was measured in both fractions using the fluorometric assay. The majority (97 %) of the GUS activity was detected in the soluble fraction, compared with 22 % when plants expressed the CPK5-16aa-GUS transgene without the G2A mutation. These results demonstrate that myristoylation is critical for the membrane binding of AtCPK5 in vivo.

### GFP fusions confirm plasma membrane localization of AtCPK5

**Fig. 5:**
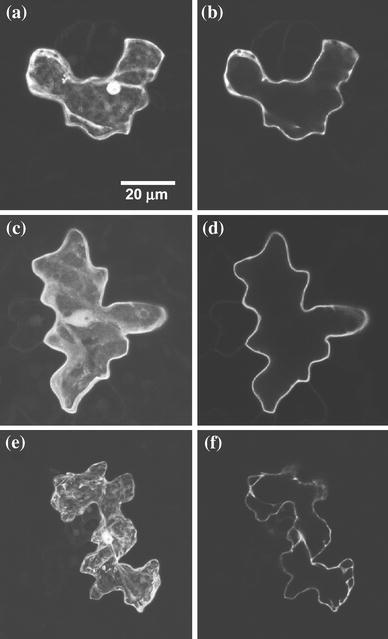
Plasma membrane localization of AtCPK5 requires glycine-2. Arabidopsis epidermal cells transiently expressing various GFP constructs were observed by confocal microscopy. **a** and **b** GFP control; **c** and **d** CPK5-16aa-GFP; **e** and **f** CPK5-G2A-GFP. **a**, **c**, and **e** are projections of 10, 30 and 26 optical sections, respectively; **b**, **d**, and **f** are single optical sections

To confirm the plasma membrane localization of AtCPK5, a construct containing the first 16 amino acids of AtCPK5 fused to green fluorescent protein was used for transient expression in Arabidopsis. pCPK5-16aa-GFP was bombarded into Arabidopsis leaves and expression in epidermal cells was examined by confocal microscopy (Fig. 5). The quantitative GUS assay data presented earlier indicated that ~80 % of AtCPK5 was plasma membrane associated and it is evident from a single optical section near the midpoint of the cell that CPK5-16aa-GFP was found at the cell periphery, corresponding to plasma membrane-localized AtCPK5 (Fig. 5d). A maximum intensity projection indicates that fluorescence was also observed in the cytosol and nucleus, likely corresponding to soluble CPK5-16aa-GFP fusion protein (Fig. 5c). Fluorescence in nuclei was observed routinely for all transgenic plants and was not unexpected since the sizes of free GFP (26.8 kDa) and CPK5-16aa-GFP (28.5 kDa) are below the exclusion limit of the nuclear pore complex (Grebenok et al. 1997). In cells expressing the non-myristoylated CPK5-G2A-GFP fusion protein, fluorescence was localized primarily in the cytosol and nucleus (Fig. 5e, f), in a pattern similar to free GFP (Fig. 5a, b). These data confirm that a substantial proportion of AtCPK5 is localized to the plasma membrane and that the amino-terminal glycine residue is required for membrane binding.

## Discussion

Calcium-dependent protein kinases have been found in all plant genomes and function in many cellular processes, including signaling in response to biotic and abiotic stresses and regulation of carbon and nitrogen metabolism (Klimecka and Muszynska 2007). In this study, we investigated the myristoylation and subcellular localization of AtCPK5 and documented the presence of phosphorylated forms of this enzyme.

In our experiments, AtCPK5 was routinely detected as a protein doublet on immunoblots using isoform-specific antibodies. The doublet was also apparent in samples containing non-myristoylated AtCPK5 and thus the change in electrophoretic behavior was not attributable to acylation. Phosphorylation can affect protein migration during electrophoresis (Peck 2006) and decreased mobility had previously been documented for a phosphorylated tobacco CDPK (Romeis et al. 2000; Romeis et al. 2001). Treatment of Arabidopsis protein extracts with alkaline phosphatase caused the disappearance of the slower-migrating form of AtCPK5, indicative of phosphorylation. In vitro calcium-dependent autophosphorylation of AtCPK5 had been documented previously (Hrabak et al. 1996) and two autophosphorylated peptides were identified (Hegeman et al. 2006). In these experiments, we cannot distinguish autophosphorylation from phosphorylation catalyzed by other kinases present in Arabidopsis or in the wheat germ extract. Some CDPKs, like tobacco NtCDPK2, are phosphorylated at specific sites in a membrane-dependent manner (Witte et al. 2010); however, phosphorylated forms of AtCPK5 were observed for both membrane-bound native AtCPK5 and non-myristoylated AtCPK5-G2A, indicating that phosphorylation was not dependent upon membrane association.

Calcium-dependent protein phosphorylation has been reported previously in plasma membrane-enriched plant cell extracts (Schaller et al. 1992; Verhey et al. 1993; Baizabal-Aguirre and de la Vara 1997; Iwata et al. 1998) and some CDPKs are known to localize to the plasma membrane (Rutschmann et al. 2002; Dammann et al. 2003; Raices et al. 2003; Chehab et al. 2004; Gargantini et al. 2006; Mehlmer et al. 2010; Kobayashi et al. 2012). We began our studies of AtCPK5 localization by fractionating Arabidopsis extracts to separate plasma membranes from intracellular membranes. Both AtCPK5 and a known plasma membrane marker were enriched in the upper fraction while a minor percentage of AtCPK5 was found in the lower cytosolic fraction, indicating that the membrane-bound AtCPK5 was primarily associated with the plasma membrane. The plasma membrane localization of AtCPK5 could facilitate activation of the enzyme by localized calcium signals generated in response to abiotic or biotic stimuli (reviewed in Kudla et al. 2010). Several plasma membrane-localized proteins, such as the H^+^-ATPase and several ion channels (Pei et al. 1996; Li et al. 1998; Rutschmann et al. 2002; Mori et al. 2006; Geiger et al. 2010), are phosphorylated in a calcium-dependent manner by CDPKs and represent potential substrates for the membrane-bound form of AtCPK5.

Membrane binding of CDPKs is proposed to be mediated by acylation. This study focused on *N*-myristoylation, an irreversible modification that occurs exclusively at an amino-terminal glycine residue (Rajala et al. 2000). The enzymology and substrate specificity of myristoyltransferases from fungi and animals have been characterized extensively (Towler et al. 1988; Rocque et al. 1993; Johnson et al. 1994; Lodge et al. 1994; Raju et al. 1995; Farazi et al. 2001; Utsumi et al. 2004). Plant *N*-myristoyltransferases are less well understood and differences between the fungal, human and plant enzymes have been noted previously (Rocque et al. 1993; Qi et al. 2000; Boisson et al. 2003; Dumonceaux et al. 2004; Pierre et al. 2007). Algorithms for myristoylation prediction such as NMT Predictor (http://mendel.imp.univie.ac.at/myristate/; Maurer-Stroh et al. 2002a, 2002b) and Myristoylator (http://us.expasy.org/tools/myristoylator/; Bologna et al. 2004) have been developed primarily using myristoylation data from fungal and animal NMTs. We have found that these algorithms do not always work well for predicting plant myristoylation sites (S. Lu, A. Argyros, and E. Hrabak, unpublished observations). NMT Predictor rejected AtCPK5 (amino terminal sequence: MGNSCRGSF) as an NMT substrate, most likely because of the arginine residue at position 6, which is rarely found in myristoylated proteins from fungi and mammals (Utsumi et al. 2004). Although peptides with arginine-6 were not myristoylated in vitro in a rabbit reticulocyte lysate (Utsumi et al. 2004), our results using wheat germ lysate clearly demonstrated that AtCPK5 was a bona fide substrate for plant NMT. Boisson et al. (2003) examined the substrate specificity of one of the two Arabidopsis *N*-myristoyltransferases in more detail and concluded that plant NMTs have relaxed substrate specificity compared to other enzymes. Subsequently, a Plant-Specific Myristoylation Predictor (http://plantsp.genomics.purdue.edu/myrist.html) was developed by Podell and Gribskov (2004) using only plant myristoylation data. Because this program was trained using our unpublished results, this website accurately predicts myristoylation of Arabidopsis CDPKs, including AtCPK5.

The majority of known CDPKs, including those from Arabidopsis and rice (Cheng et al. 2002; Hrabak et al. 2003; Ye et al. 2009), contain predicted amino-terminal myristoylation sites, although myristoylation has been demonstrated experimentally for only a small group of CDPKs. Known myristoylated CDPKs are: Arabidopsis AtCPK2, AtCPK3, AtCPK6, AtCPK9, and AtCPK13 (Lu and Hrabak 2002; Boisson et al. 2003; Benetka et al. 2008; Mehlmer et al. 2010), rice OSCPK2 (Martin and Busconi 2000), tomato LeCPK1 (Rutschmann et al. 2002), potato StCDPK1 (Raices et al. 2001), *Mesembryanthemum* McCPK1(Chehab et al. 2004), and *Cucurbita* CpCPK1 (Ellard-Ivey et al. 1999). Here, we showed that AtCPK5 is myristoylated on its amino-terminal glycine residue and localized to the plasma membrane. The first 16 amino acids of AtCPK5, which contain the myristoylation consensus sequence, were sufficient to direct plasma membrane targeting of both GUS and GFP. A G2A mutation abolished both myristoylation and in planta membrane association of AtCPK5, demonstrating that myristoylation was absolutely required for membrane binding. Plasma membrane localization has been demonstrated for other CDPKs, including AtCPK9, AtCPK13, LeCPK1, StCDPK1 and StCDPK5 (Rutschmann et al. 2002; Raices et al. 2003; Kobayashi et al. 2012) but other membrane locations are possible. Myristoylated AtCPK2 is targeted to the endoplasmic reticulum (Lu and Hrabak 2002), AtCPK3 is nuclear and perinuclear (Dammann et al. 2003; Mehlmer et al. 2010), and McCPK1 localizes to multiple membranes (Chehab et al. 2004). Thus the amino terminal region of different CDPKs could be used for as targeting signals to direct proteins to specific membranes.

Myristoylation is unlikely to be the sole determinant of CDPK membrane binding because the hydrophobicity of myristate is not sufficient to maintain long-term membrane association (Peitzsch and McLaughlin 1993; McLaughlin and Aderem 1995). The first 16 amino acids of AtCPK5 include a potential palmitoylation site at cysteine-5. Preliminary experiments indicate that Cys-5 is palmitoylated in planta (A. Argyros & E. Hrabak, unpublished data). Palmitate has about tenfold greater membrane binding affinity than myristate and palmitoylated proteins are invariably membrane associated (Resh 2004). The current model for dual acylation, the kinetic bilayer trapping hypothesis of Shahinian and Silvius (1995), posits that the weak hydrophobicity provided by myristate allows proteins to transiently sample a variety of cellular membranes where specific palmitoyl transferases reside. Conversely, mutation of the myristoylation site prevents transient membrane binding and subsequent cysteine palmitoylation (Degtyarev et al. 1994; Hallak et al. 1994; Wilson and Bourne 1995; Martin and Busconi 2001). Thus, even though wildtype AtCPK5 may be myristoylated and palmitoylated and the palmitoylation site is still present in the G2A mutant, we predicted complete loss of AtCPK5 membrane association when myristoylation was prevented, which is what we observed.

Our results show that AtCPK5 is plasma membrane localized and that the amino terminus of the protein is required for correct localization. Myristoylation of the amino terminal glycine residue of AtCPK5 was critical for membrane binding. The subcellular location of AtCPK5 will be useful for deciphering its substrates and role in plant signal transduction pathways.
